# An in-depth investigation of the causes of persistent low membership of community-based health insurance: a case study of the mutual health organisation of Dar Naïm, Mauritania

**DOI:** 10.1186/s12913-017-2419-5

**Published:** 2017-08-07

**Authors:** Maria-Pia Waelkens, Yves Coppieters, Samia Laokri, Bart Criel

**Affiliations:** 1 Université libre de Bruxelles (ULB), School of Public Health, 808 Route de Lennik, 1070 Brussels, Belgium; 2 Université libre de Bruxelles (ULB), School of Public Health, Health Policy and Systems – International Health, 808 Route de Lennik, 1070 Brussels, Belgium; 3 Tulane University, School of Public Health and Tropical Medicine, Global Community Health and Behavioral Sciences, 1440 Canal Street, New Orleans, LA 70112 USA; 4 Department of Public Health – Equity & Health, Institute of Tropical Medicine, Nationalestraat 155, 2000 Antwerp, Belgium

**Keywords:** Community-based health insurance, Mutual Health Organisations, Implementation, Universal health coverage, Sub-Saharan Africa

## Abstract

**Background:**

Persistent low membership is observed in many community-based health insurance (CBHI) schemes in Africa. Causes for low membership have been identified and solutions suggested, but this did not result in increased membership. In this case study of the mutual health organisation of Dar Naïm in Mauritania we explore the underlying drivers that may explain why membership continued to stagnate although several plans for change had been designed.

**Methods:**

We used a systems approach focussed on processes, underlying dynamics and complex interactions that produce the outcomes, to delve into 10 years of data collected between 2003 and 2012. We used qualitative research methods to analyse the data and interpret patterns.

**Results:**

Direct causes of stagnation and possible solutions had been identified in the early years of operations, but most of the possible solutions were not implemented. A combination of reasons explains why consecutive action plans were not put into practice, showing the complexity of implementation and the considerable management capacity required, as well as the challenges of integrating a novel organisational structure into exiting social structures.

**Conclusions:**

For any CBHI project aiming at high membership, skilled professional management seems essential, with capacity to question and adapt routine procedures and interpret interactions within the wider society. Countries that include community-based health insurance in their strategic plan towards universal coverage will have to pay more attention to management capacity and the minutiae of implementation.

## Background

Community-based health insurance (CBHI) refers to voluntary, non-profit health insurance, organised at local level where state provision or formal health insurance do not provide protection against the cost of illness. CBHI applies the principles of insurance, i.e. resource pooling, prepayment and risk-sharing, and negotiation with other partners in the health system to improve access to care, financial protection and responsiveness of health services [1]. A mutual health organisation (MHO) is the type of CBHI scheme most common in West Africa, governed by its members.

CBHI has been introduced in many African countries in second half of the 20th century [2]. Especially in the 1990s and 2000s there was a rapid expansion, when CBHI was considered a stepping stone towards national health insurance [3]. Results, however, did not match expectations, and the initial enthusiasm of the international community to support CBHI decreased. Yet before deciding to reject CBHI as a health financing strategy, it seems reasonable to verify whether its implementation received sufficient attention. Moreover, many governments included CBHI in their current strategies towards universal health coverage, particularly to reach the informal sector, which further advocates for an in-depth examination of implementation processes and practices, and whether and how they can be improved.

### Membership: a performance indicator for CBHI

Because affiliation is voluntary, membership is a yardstick of performance of CBHI: health insurance that offers interesting benefits will attract members; low membership should prompt investigation why the health insurance scheme does not attract more members.

The main indicators for measuring membership are population coverage or the proportion of the target population that is insured, the growth rate or the number of members compared to the previous year, the renewal rate or the proportion of beneficiaries who renew their subscription, and the proportion of members amongst patients attending health services.

When calculating population coverage, the figure that matters is not the number of registered beneficiaries, but that of active beneficiaries, i.e. beneficiaries who are up-to-date with premium payment and are entitled to benefits. The renewal rate indicates the number of beneficiaries eligible for renewal at the start of the year who did renew their subscription. It is normally an indicator of members’ satisfaction and the capacity of the health insurance scheme to retain members. Alternatively, this question can be measured by its opposite, the “drop-out” rate. The proportion of members amongst patients is an indicator of the power the members’ organisation may have to influence health care provision.

From the very early to more recent reviews of the evidence, low membership is persistently observed in CBHI schemes in sub-Saharan Africa [2, 4–6]. Many causes contributing to low membership have been identified. They include contextual factors such as affordability of contributions, understanding and accepting the principles of insurance, distance to health facilities and the quality of health care they offer, trust in scheme management and care provider, the existence of an appropriate legal framework and financial support, and internal factors related to CBHI scheme’s organisation and management such as provision of interesting benefit packages, response to expectations, information of the target population, design issues such as the unit of enrolment and the timing of collecting contributions, technical and management capacity [1, 5–13].

Recent studies continue to focus on the determinants of membership both in terms of reasons for affiliation [14–18] or reasons for drop-out [16, 19–21]. There are few follow-up studies, however, that check whether recommendations to improve performance were implemented, to which outcome, and why or why not. This implementation is the focus of our case study of the MHO Dar Naïm in Mauritania. We present the findings from the perspective of low membership.

### Creation of the MHO of Dar Naïm

**Fig. 1:**
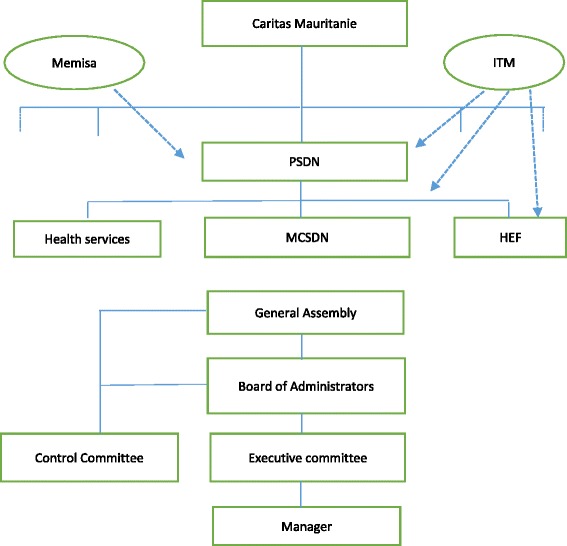
Organigram of MCSDN and support organisations

Dar Naïm is a suburb of Nouakchott, the capital of Mauritania. It was created in 1989 after an influx of rural populations, and remains one of the poorer suburbs of Nouakchott where new migrants settle first. In 2007 about 39% of households gained their income in the informal sector, 26% were without work, 17% were government employees, 12% private sector workers and 6% pensioners [22]. The government appointed the NGO Caritas Mauritanie^1^ to organise health care in Dar Naïm. Since 1990, its “Projet Santé Dar Naïm (PSDN)^2^” is the main provider of first line health care in the area. Inspired by examples in neighbouring Senegal, the management team of the PSDN proposed to establish an MHO to improve financial access to health care. All preparations, feasibility study, organisational structure, management procedures and tools followed the guidelines of the Strategies and Techniques against Social Exclusion and Poverty programme (STEP) of the International Labour Organisation (ILO), later described in several guides [23–25]. The overall design of the scheme was that of a Mutual Health Organisation (MHO), characterised by ownership by its members and governed by a General Assembly, Board of Administrators, Executive Committee and Control Committee [26, 27] (Fig. 1). This model was selected with the understanding that its organisational structure would “strengthen the capability of the community to gain control over the factors and decisions that affect their health”, an objective of the MHO project [28]. Funding for set-up and management was provided by the Belgian organisation Memisa^3^ for a period of 10 years.

Preparations started in August 2000, the feasibility study from initial training to restitution of results took place from May to November 2002 [18]; and the Mutual Health Organisation of Dar Naïm (Mutuelle communautaire de santé de Dar Naïm - MCSDN) was officially created on January 11th 2003. Payment of the first claims started on July 1st 2003, for those members who had paid premium during a waiting period of 6 months.

The PSDN had divided Dar Naïm in seven, later eight, zones, and decentralised “*Bureaux de zones*” were established with each an Executive Board of voluntary delegates. Only the manager of the MHO is remunerated. The organisational structure and the attributes of each function, as well as membership conditions, benefits and functioning of the MHO are laid down in the Statutes, composed of a Constitution and Rules and Regulations [29].

### Membership procedures

Membership is geographical. All residents of Dar Naïm, without exclusion, can subscribe to the MHO. There is no specific period of enrolment: members can register at any time of the year. The household is the unit of affiliation. Membership is voluntary, but affiliation of all dependents is automatic: to limit adverse selection, a household has to subscribe with all its members and pay contribution for each dependent. Members formed units of ten households and elected their delegate who represents the unit in the General Assembly.

Each household paid a membership fee of 200 Mauritanian Ouguiya (MRO) (0.71 EUR) from 2002 to 2006 and of 300 MRO (0.97 EUR) since 2007. A membership booklet identifies beneficiaries when seeking care. Premium was 50 MRO (0.18 EUR) per person per month in 2003 and increased to 80 MRO (0.21 EUR) or 840 MRO per year in 2012, following inflation and higher charges for care. Membership is terminated when due premiums are not paid during six consecutive months.

### Persistent low membership

All stakeholders involved in the MHO project of Dar Naïm had expected a rapid expansion of the MHO and agreed that it had been set up in favourable conditions. The feasibility study had indicated that a large majority of the population was able and willing to pay [28]. There was a dynamic tissue of cooperatives, associations and microinsurance initiatives. Many referred to the Koran to support their positive opinion of the creation of a mutual health insurance scheme. Health care services were well attended: for the latest episode of illness, 71% sought care in the formal health sector, while 18% stayed at home expecting spontaneous recovery, 8% preferred auto medication and 3% sought care with a traditional healer [28]. The PSDN that launched the initiative was well known and trusted, the project had the support of health authorities and local authorities, and sufficient funding was secured to sustain set-up, management and monitoring and evaluation for a 10-year period. But 1 year into operations the dwindling number of active beneficiaries endangered the viability of the scheme. In September 2003, only 828 out of 9750 registered beneficiaries (8.5%) were up-to-date with payment. The PSDN and its supporting organisations Caritas Mauritanie and Memisa requested technical assistance from the Institute of Tropical Medicine (ITM^4^). In December 2012, after 10 years of operation and repeated attempts to remedy those determinants that seemed to cause stagnation, membership continued to stagnate.

This deadlock prompted us to revisit our material gathered during a 10-year period from 2003 to 2012 to uncover factors underlying the stagnation of the MHO of Dar Naïm and especially to understand what withheld implementing change. The findings may be food for thought for many other CBHI schemes where membership remains low even when direct causes for low membership have been identified.

## Methods

### A ten-year action research process

Data for our current, retrospective study were collected over a 10-year period from 2003 to 2012 in the context of action research [30, 31] that accompanied the programme to guide improvement of performance, with a research agenda defined by the problems that managers faced in daily practice. A first programme evaluation in December 2003 was followed by six more, the latest in December 2012. Each evaluation visit took about 14 days. It started with measurement of outcomes against objectives, based on performance indicators used in the routine monitoring system, discussion of achievements and challenges encountered in implementing the latest action plan, and identifying particular queries that needed looking into. Proposed solutions were often formulated as hypotheses to be tested, the results evaluated and new solutions proposed in an iterative process that was part of the management cycle. The research process was participatory. The principal investigator was not an external observer, but also an actor in the process.

From 2005 onwards, in addition to the evaluation process focused on local needs, we used a standardized protocol for data collection, a checklist of essential factors to look into during each evaluation. Its underlying aim was to collect similar data in the various CBHI programmes in which we were involved to prepare cross-case comparison of performance of CBHI and of the contextual conditions and implementation processes that influence outcomes. Questions regarding performance were classified following the major goals of CBHI we had identified [32]: health system goals (1. access to health care services, 2. contribution to equitable health financing, 3. impact on service delivery), economic goals (4. protection of household assets) and socio-political goals (5. inclusiveness, 6. empowerment of members) (Box 1). Questions related to the process to achieve these goals were organised into two axes of analysis: viability of the scheme and management.

The routine monitoring system of the MHO of Dar Naïm is the source of quantified data on membership, financial situation and use of health services. The database of the PSDN is the main source of information on utilization and cost of health services. Data from hospitals and the health district were at the time of data collection either inaccessible or incomplete. Two comprehensive surveys were carried out in the course of the programme. The first, the feasibility study carried out in 2002, provides information on socio-economic background, willingness to participate, perceived priorities in service provision, ability to pay, health service utilization and available health services at the start of the project [28]. The second was a population survey carried out in December 2007 in which 1200 household heads were interviewed to understand their opinion about membership and the future of the MHO [22]. Ad hoc studies were carried out to answer specific questions, such as cost and quality assessments of health facilities, in-depth interviews and group discussions with community members, with actors involved in the management of the scheme and with key persons in local and national institutions. Their results are included in the evaluation reports that are the main source of the current data revision.

### The current data revision

To gain more understanding of why things happened as they did, the material gathered during this 10-year research process was taken up again to analyse the data, with hindsight, using a systems approach that focused on the processes, the underlying dynamics and the complex interactions that produce the outcomes [33]. The system under review is the MHO itself, with its organisational structure, design and management. It functions in a societal, legal, economic, cultural, political and administrative environment, with privileged interactions with the health sector. Members and CBHI scheme leaders are at the same time part of the wider society, which directly influences their behaviour within the scheme. We had to consider both the system’s internal processes of transformation and its interactions with its environment.

### Organising data

Framework analysis [34, 35] offered a structured approach to organise the huge amount of data and examine a vast diversity of questions related to the performance of CBHI. We retained the six areas of performance of our protocol for data collection and combined its determinants of performance with those examined in influential reviews of the effectiveness of CBHI [1, 5, 36–40] and manuals of CBHI evaluation [25, 41, 42].

The resulting framework consists of a comprehensive set of indicators and questions arranged under twenty headings (Table 1). The first themes describe baseline and creation, providing relevant information to understand the local context and information against which developments can be compared. The subsequent themes each concern an angle from which the performance of CBHI can be examined.

**Table 1:** Framework for organising data and analysis

Areas of performance:
1. Access to health care services
2. Contribution to equitable health financing
3. Impact on service delivery
4. Protection of household assets
5. Inclusiveness
6. Empowerment of members.
Determinants of performance:
1. Creation: the objectives formulated by each stakeholder and the process that led to the launching of the CHI scheme.
2. Environmental profile: background information on socio-economic conditions, the health system, health service delivery and quality of services, and health financing.
3. Preparedness: readiness of the national administrative, legal and financial system, of the health sector and of the target population to integrate community health insurance.
4. Resource mobilisation describes design and implementation related to premium, co-payment and subsidies.
5. Marketing and communication
6. Financial management: the administrative functions of budgeting and bookkeeping.
7. Financial viability: financial results and specific indicators measuring financial viability of CHI.
8. Managing risks: strategies to manage adverse selection, over-consumption, provider’s prescription, and fraud.
9. Financial protection: risk-spreading between healthy and sick, prepayment to reduce direct payment at the time of illness and measures to reduce the overall bill.
10. Premium calculation: how were benefits’ package and premium calculated
11. Benefits: the package of services, conditions for accessing benefits, evolution of the package over time.
12. Membership: rules and regulations, membership statistics and reasons for affiliation and drop-out.
13. Social inclusion: strategies and activities for inclusion of vulnerable groups.
14. Utilisation: utilisation figures of members and non-members; health seeking behaviour.
15. Provider payment: rules and management practices related to claims’ payment.
16. Health care provision: health care providers, quality of care, relationship between CHI scheme and health care providers, strategies and action to influence provision of care.
17. Stewardship: legislation, government involvement.
18. Governance & decision-making: organisational structure, interactions within the scheme.
19. Role table: support to get insights in interactions.
20. Empowerment: ‘empowerment in action’ describes the practices of community participation within the MHO; ‘empowerment as a result’ explores whether the organisational structures of the MHO influence power relationships in matters of health and society.

The original data were rearranged and summarized within the part or parts of the framework to which they relate. The same data can be classified under several headings, where they are interpreted from another viewpoint. Within each theme, classifying followed a sequence from objectives to outcomes (Objective → Design → Implementation → Outcome), with a separate category for interactions between actors. Quantified data from the routine monitoring systems were compiled in tables providing a 10-year overview and classified under the appropriate headings.

### Analysis

This charting process involved a first level of abstraction and synthesis that paved the way for finding associations and explanation. Since our aim was to find meaning, our exploration needed to interpret patterns rather than simply detect them, and find those interactions that drive the system. We used grounded theory [31, 43] as the principal research method to analyse the data and interpret patterns: letting data speak for themselves to organise, classify, generate hypotheses and build a theory from the bottom that explains the behaviour of the system. This was first done on a theme by theme basis, from the particular viewpoint of the theme heading, considering for each theme external factors influencing the process, internal factors and stakeholder interactions. Quantified data served the sole purpose of describing outcomes, without further quantitative analysis. Next, reading across themes highlighted underlying facts, events and processes that seem to influence outcomes in various themes. These emerging patterns generated theoretical understanding, formulated as hypotheses that explain what held back the performance of the MHO of Dar Naïm. They will be the main issues selected for subsequent cross-case comparison with similar studies of other CBHI schemes, to generate a higher-level grounded theory.

The structured analytical process used to organise data is not necessarily conflicting with grounded theory approach [35]. Each thematic heading can be seen as an individual research question, within which analysis is done using the constant comparison method of a grounded approach: testing first one element to another, then confronting emerging theoretical constructs to new data, and theoretical ideas across themes.

## Results

The findings reported here are a selection of determinants of persistent low membership of the MHO of Dar Naïm, illustrated by events that seem most telling to substantiate our path to conclusions.

### Main membership figures

In June 2003, when claims payment started, there were 9655 registered beneficiaries for a population of 59,486 (Table 2); a population coverage of 16.2%. One year later, however, 6188 beneficiaries had been terminated because they had not paid contributions for more than 6 months. Since then, the average number of active beneficiaries is around 3086. At its peak in 2008 and 2009, 7% of the population of Dar Naïm were active beneficiaries. In 2012, 3233 active beneficiaries represented 3.8% of the population that was then estimated at 85,000. Not all members were self-paying. In 2005, the PSDN had started a Health Equity Fund (HEF) to improve access to health care for the poorest [44]. By 2012, the HEF paid the contributions of 23% of all active beneficiaries of the MHO.

**Table 2:** Number of active beneficiaries entitled to benefits over time^a^

	2002	2003	2004	2005	2006	2007	2008	2009	2010	2011	2012
Registered beneficiaries	8869	9655	4894	6223	6696	7207	7054	6234	5672	4950	6311
Active beneficiaries		2281	1475	2523	3058	2481	4501	4763	3946	2598	3233
Estimated population	64,800	59486^b^	60,000	62,000	64,000	65,000	65,500	69,801	70,000	85,000	85,000
Effective coverage (%)		4%	2%	4%	5%	4%	7%	7%	6%	3%	4%
Beneficiaries HEF				51	200	293	452	510	598	674	736
% self-paying beneficiaries		100%	100%	98%	93%	88%	90%	89%	85%	74%	77%
Growth Ratio^c^ (%)			−35%	71%	21%	−9%	81%	6%	−17%	−34%	24%

^**a**^ The membership figures provided by the monitoring system of the MHO of Dar Naïm contain inconsistencies. Figures provided here are based on meticulous comparison of all available data

^b^ A survey done by the PSDN under supervision of the “*Office National de la Statistique*” in September 2003

^c^ (Number of insured Current year – Number of insured previous year) / Number of Insured previous year

The average drop-out rate, calculated on basis of terminated memberships, is 26% (Table 3). This does not inform about long-term retention of remaining members. It could as well be that they remain in the MHO for 1 or 2 years, then drop out, register again years later, etc.

**Table 3:** Drop-out rate

	2003	2004	2005	2006	2007	2008	2009	2010	2011	2012
Registered beneficiaries on January 1st	8869	9655	4894	6223	6696	7207	7054	6234	5672	4950
New beneficiaries in the six first months^a^		735	1017	795	841	4215	652	648	774	685
Total beneficiaries on June 30th		10,390	5911	7018	7537	11,422	7706	6882	6446	5635
Terminated beneficiaries on December 31st		6188	705	1568	1741	4851	1828	1497	2152	0
Drop-out rate		0.60	0.12	0.22	0.23	0.42	0.24	0.22	0.33	0.00

^a^ Membership is terminated when premium is not paid during 6 months

The proportion of MHO members amongst the patients attending health services was measured in the four health facilities of the PSDN where most members attend. MHO members are only a minority: they account for 7% of consultations, for 2% of births, and 5% of the revenues (Table 4).

**Table 4:** Proportion of MHO members among attendants of the PSDN health structures

	2004	2005	2006	2007	2008	2009	2010	2011	Total
% of consultations (curatives, pre- and postnatal^a^) by MHO members									
Consultations by MHO members	1119	1696	2940	3562	4353	5536	4243	2691	26,140
Total consultations	35,501	37,292	41,668	46,777	51,304	56,636	54,429	58,895	382,502
% MHO	3%	5%	7%	8%	8%	10%	8%	5%	7%
% of deliveries by MHO members									
Deliveries by MHO members^b^	21	38	50	23	44	98	102	85	461
Total deliveries	1876	1992	2056	2267	2535	2700	2408	2580	26,228
% MHO	1%	2%	2%	1%	2%	4%	4%	3%	2%
Contribution^c^ of the MHO in cost recovery of the PSDN health structures									
% MHO					5%	6%	5%	3%	5%

^a^ The monitoring system of the MHO does not separate curatives, pre and postnatal consultations

^b^ Incomplete for 2008 and 2009

^c^ Co-payment paid by members is not included in the income from MHO members

### Technical solutions for deficiencies in design and management

During a first evaluation of the MHO that took place in December 2003, a range of difficulties contributing to the low contribution rates were identified (Table 5). These difficulties were not related to external conditions, but to internal management. Some required changes in the original design, others required different management approaches or redefining roles and relations between actors. Possible solutions were extensively discussed with key actors during this and consecutive programme evaluations, but planned actions were only very partially implemented. This implies that beneath the identified causes for stagnation and their technical, straightforward solutions, there is a second layer of obstacles that prevent implementing change. The following examples illustrate underlying obstacles to action.

**Table 5:** Identified problems and solutions, December 2003

Problem	Discussed solutions	Implementation
Ineffective procedures for premium collection:	Comprehensive revision of procedures for premium collection:	
- Monthly collection	- Annual collection - Close follow-up of due renewals	- Option from 2008 onwards - No close follow-up
- Transport to be paid by volunteers	- Monthly tour of all zones by the manager	- Successfully executed during several months, then interrupted
- Many delegates are discouraged by demanding tasks	- Replacement of inactive delegates - Transfer of tasks from volunteers to the paid manager	- No replacements - No explicit transfer of tasks; in practice some tasks were taken on by the manager
- Mismanagement of funds by some delegates	- Enactment of sanctions as stipulated in the Statutes	- No sanctions; individual discussions by the President of the MHO
Complicated procedures for proving entitlement when seeking care	Abolishment of the ‘guarantee letter’ proving entitlement and timely distribution of lists of active beneficiaries to care providers	- Abolished in 2007 - Timely distribution of lists from 2008 onwards
Poor understanding of multiple and complicated rules and regulation	- Better information of leaders, delegates and members about procedures, rights and obligations - Simplification of procedures where possible	- Several training sessions for scheme leaders and delegates - Regular information campaigns
Disinformation by recruiters with the aim to register high numbers	New information campaign; open exchange with members about the initial disinformation by some	Dynamic and candid information campaign in 2004
Distrust of members resulting of insufficient information, disinformation and mismanagement of funds	New information campaign; transparency; better communication; promotion of participation and ownership	Several information campaigns; no change in communication style, participation and feeling of belonging and ownership
No perception of belonging and ownership by members	Regular meetings with members in each zone to discuss health subjects of general interest	- Done during several months - Successfully done in the zone of Zaatar
Poor performance of delegates in positions of responsibility, who had expected personal rewards	- Replacement of inactive delegates by motivated candidates - Abolishment of *bureaux de zone*	- No replacement of delegates - No explicit changes in the role of the *bureaux de zone*
Exclusion of the poorest and large households; disinterest of wealthier households	- Creation of an equity fund - Redefinition of ‘the household’ - Discounts for large households - Targeted information campaigns - Advocacy with municipal administration to integrate the MHO among other public services	- Done in 2005 - Spontaneous evolution towards the nuclear family - No discount for large households - No campaigns to attract the wealthier - No rapprochement with municipal administration
Inaction of scheme leaders when faced with unexpected problems	- Responsive management aiming at problem solving and members’ satisfaction - Continuing education	- Leaders tried to improve implementation of initial strategies; no change in management style - Frequent training workshops until 2008

### Procedures for proving entitlement: a successful change

To prove entitlement to benefits when seeking care, patients needed to present their membership booklet in which their picture appeared and a ‘guarantee letter’ issued by the *bureau de zone*, proving that their household was up-to-date with contributions. Obtaining the document from the *bureau de zone* was not practical. The bureau may be far away from the health facility or the person who issued the letters could not be found. When finally seeking care without the guarantee letter, members were denied benefits. Frustration and disappointment made them give up monthly payment.

A simpler way to check entitlement was to abolish guarantee letters and distribute lists of active members to the health facilities, where the staff directly checks entitlement with the list of names.

However, the Board of Administrators was reluctant to abolish the guarantee letter, partly because they were not inclined to make changes to the original design, partly because it would reduce the role of the *bureaux de zone* and might offend the local leaders who managed them. All community leaders had been given a position of responsibility in the MHO to safeguard social harmony. Not antagonising them was a predominant motive underlying decisions of the Board of Administrators. However, the manager was convinced that simplified procedures for entitlement were essential to avoid frustration of active beneficiaries seeking care. Owing to her persistent efforts the guarantee letter was abolished in 2007 and timely distribution of the lists was routinely achieved in 2008. Since then, the new procedures to prove entitlement are accepted by all and few even remember the previous system that leaders wanted to maintain.

### Procedures for premium collection: a partial but insufficient change

Premium collection, as initially designed, involved many people: delegates collect monthly contributions from their unit of ten members and transfer it to the treasurer of the *bureau de zone* who in turn transfers the moneys to the central MHO office. In practice, many delegates ceased to collect monthly contributions of their unit members, because it involved more efforts than they were willing to bear on a voluntary basis. The *bureaux de zone* did not perform as expected and three did not function at all. Thus, members who wanted to pay their contribution did not know where to go.

An alternative that worked well was direct payment by delegates to the manager, who went monthly to each zone to collect contributions, thus cutting out intermediate steps between members and the bank account of the MHO, avoiding delays, solving the problem of transport costs for volunteers, and collecting contributions where the *bureaux de zone* were dysfunctional. However the monthly tour by the manager was interrupted, mainly because it devalued the role of the *bureaux de zone*.

Annual payment, which would greatly simplify premium collection and was affordable for most members, was initially rejected by the Board of Administrators because they did not know any other scheme that did so. It was later accepted when a survey done at the end of 2007 revealed that interviewed ex-members’ (176/1200, 15%) main advice was to improve the system for premium collection, and that current members (349/1200, 29%) were largely in favour of annual payment, which was most interesting for informal sector workers with irregular income patterns [22]. After the survey, annual payment was accepted and progressively made compulsory for new members. By 2012, most households paid an annual contribution, but not all problems were solved. Follow-up of renewals was not organised. Those who forgot to renew were not traced for reminding and their membership was terminated after 6 months of arrears.

Instead of focusing on measures that would improve retention of existing members, the Board of Administrators prioritised regular sensitisation campaigns to attract new members. Most recruiters, in their enthusiasm to bring in high numbers, registered households that had paid the membership fee only, without considering subsequent premium payments. Households that had paid a membership fee were usually not re-contacted, often did not receive membership booklets, did not pay regular contributions and their membership was terminated at the end of the year. Between 2002 and 2012, a total of 5495 new members covering 26,841 beneficiaries had been recruited and registered. Then again, 4209 members covering 20,530 beneficiaries did not pay their premium and were terminated.

In 2012, the MHO leaders recognised that discontinued premium payment was still a major obstacle to expanding membership, but remained reluctant to fundamentally review procedures for premium collection or other measures to retain members.

### Maintaining dialogue with members: a successful initiative not extended

Where the delegate and/or the *bureau de zone* stopped functioning, it was difficult for ordinary members to maintain contact with the MHO. To restore communication with members, the manager started organising meetings in each zone. When this initiative was stopped to reduce costs, she decided to organise members’ meetings in the zone of Zaatar for which she held the *bureau*. Incidentally, the number of members of this zone increased from 5 households in 2005 to 148 households in 2012. Zaatar is the only zone where the number of active beneficiaries steadily increased over the years (Table 6). There is, for example, one unit of ten women who formed a Rotating Savings and Credit Association or ROSCA. Each month the one who receives the collected sum uses it to pay her annual premium. All are fairly well informed of the benefits, and when in doubt, they know where to get information. These are satisfied and motivated members. They attribute the positive result in their area to proper information of members, regular meetings, listening and adequate response to their problems, and mutual aid within the unit when necessary. This positive experience was not extended to other zones. Their children, once independent, become members.

**Table 6:** Average active beneficiaries per zone

Zone	2004	2005	2006	2007	2008	2009	2010	2011	2012
Dar Barka			274,5	248,7	434,4	360,3	200,8	72,1	71,5
Dar Salam	359,2	871,7	488,75	439,8	411,3	432,0	439,8	387,3	496,5
Eolienne	57,9	233,2	172,7	179,8	183,1	161,5	135,2	178,8	330,9
Hay Saken	210,1	473,5	290,8	295,9	360,3	322,8	112,3	113,6	89,3
Maison des jeunes	40,8	157,5	755,9	202,2	273,1	591,1	556,3	473,3	449,8
Tab Salam Diam	339,2	618,2	755,9	847,6	1206,9	1658,1	1118,7	640,1	735,6
Tensoueilem	63,2	116,3	98,0	75,3	97,9	108,8	89,7	73,9	79,1
Zaatar	52,8	112,2	142,1	191,6	261,2	387,3	432,2	467,5	629,5
HEF^a^							59,8		
Total	1123	2582,6	2401,1	2480,8	3212	4021,9	3144,6	2406,5	2882,2

^a^For other years beneficiaries of the Health Equity Fund are included in the numbers per zone

### Cost containment: an essential change systematically ignored

In 2003 the fixed reimbursement of 10,000 MRO (36.8 €) for hospitalisation had been sufficient to cover patients’ expenses in the close-by Cheikh Zayed Hospital. Hospital bills increased dramatically in subsequent years. Also medicines to be bought in private pharmacies that were routinely prescribed by hospital physicians were a huge burden especially for patients with chronic disease. Reimbursement for hospitalisation was increased to 20,000 MRO in 2005 and 30,000 MRO in 2011, but by then only covered a fraction of real expenses that were often over 100,000 MRO. Reimbursement of medicines bought in private pharmacies was introduced in 2005, but was in 2007 reduced from 50 to 30% for chronic diseases to remedy a deficit. In 2010, it was interrupted altogether until the balance between income and expenses was positive again. Each time, the reduction of benefits was followed by exit of disappointed members.

Cost containment strategies were included in the action plans from 2007 onwards. They involved documenting the real cost of hospitalisation and presenting this evidence to hospital management, discussing rational prescription and use of generic medicines with hospital physicians and members, and establishing a restrictive list of medicines covered by the MHO that included cheap generic medicines for chronic conditions available at first line. First steps were taken several times but the efforts were not continued. For example, in December 2009 the MHO carried out a survey in Cheikh Zayed Hospital in which patients of various wards were interviewed daily until their departure and asked to detail all their expenses. The results of the survey were not compiled, and the survey forms could later not be found. No list of covered medicines was introduced, nor was there discussion of prescription with hospital physicians.

Another realistic option to reduce members’ expenditure was to make advantage of available subsidies. Public hospitals in Mauritania receive a substantial government subsidy to pay for health care of the poor. Applicants can approach the hospital’s social services or request a certificate with the Department of Social Affairs (*Direction des affaires sociales* (DAS)). In 2007 the Health Equity Fund made an arrangement with the social services of Cheikh Zayed Hospital whereby 70% of the hospital bill for its members were paid by the government fund [44]. The MHO did not follow this example, nor did they engage the procedures for obtaining a certificate for free hospital care from the DAS.

In 2009 the National Health Insurance Fund (CNAM) opened another door. Its director proposed CNAM coverage for hospital care for the members of the MHO in return for a contribution to be negotiated. Finally, several international institutions present in Mauritania may have welcomed the MHO as a rare initiative for social protection through which to channel their funds for universal coverage. They could, for example, have been interested in a pilot project looking into how best organise CNAM coverage for hospital care for MHO members, or how best cover people with chronic disease. Again, first steps were taken but no sustained efforts made to take advantage of these genuine opportunities.

### Decreasing the benefits to balance the books

Since no measures were taken to reduce expenses, how did the MHO deal with the ever increasing health care charges?

The benefits’ package selected in 2003 had been limited. Once members had experienced health insurance, in 2004 the General Assembly asked for its expansion. The feasibility of changes in benefits was calculated by the PSDN support team and laboratory examinations, ambulatory hospital care and later caesarean section were included. Emboldened by the positive reactions of members, the Board of Administrators continued to expand the package in 2005 with reimbursement of medicines bought in private pharmacies and dental care, without, however, calculating what this would mean in terms of premium.

Substantial deficits in 2006 and 2007 required measures to restore financial viability. Premium was increased from 600 to 720 MRO (1.69 to 1.98 EUR) per year in 2008 and to 840 MRO (2.25 EUR) in 2012, but this did not fully compensate for price increases. Members agreed with the premium adaptations and, most being involved in micro-businesses, would have perfectly understood an annual adaptation of premium following inflation. There was also scope to do so: in 2007 average health expenses of non-members were 6214 MRO per person per year, when the annual premium was 720 MRO per person [22]. The MHO leadership, however, preferred to keep the premium low as a way to attract members. The MHO leadership, however, preferred to keep the premium low as a way to attract members.

If premium does not follow fee increases, other sources have to be found to maintain financial solvency. The main mechanism practiced to avoid deficits was to reduce benefits: either co-payment was raised, or coverage of services was reduced, both resulting in ever-increasing out-of-pocket expenses and declining financial protection. For first line curative care, co-payment was increased from 50 MRO in 2004, to 100 MRO in 2009 and 200 MRO in 2010. In 2012, out-of-pocket spending for first line consultations represented 40% of the bill (Table 7); more than 50% in the health post of the PSDN that had always striven to provide quality care at the lowest price. As mentioned before, reimbursement of medicines for chronic care bought in private pharmacies was reduced and also coverage of hospital care had become insubstantial. Increasingly members expressed the opinion that benefits were not worth affiliation.

**Table 7:** Proportion of consultations fees paid out-of-pocket in 2012

First line health facility	average Fee	co-payment	% co-payment
PSDN			
Tab Salam Diam	575	200	0,35
Tab El Avia	309	200	0,65
Tab El Khair	369	200	0,54
Tab Teissir	345	200	0,58
Subtotal PSDN	466	200	0,43
Government			
Tensouïlim	806	200	0,25
Teyarett	926	200	0,22
Other			
Tab Rava	559	200	0,36
Total	495	200	0,40

Table 8 gives an overview of the evolution of the benefit package and Table 9 summarises the benefits’ package as it was in 2012.

**Table 8:** Evolution of benefits’ package and financial coverage in the MCSDN: Summary 2003–2012

	Payments by MCSDN								
	2003	2004	2005	2007	2008	2009	2010	2011	2012
First line health facilities	4 of PSDN	5	7	7	8	8	8	8	8
Curative consultations	50%	Above 50 MRO	Above 50 MRO	Above 50 MRO	Above 50 MRO	Above 100 MRO	Above 200 MRO	Above 200 MRO	Above 200 MRO
Antenatal care	50%	50%	75%	75%	75%	75%	75%	75%	75%
Delivery	75%	75%	75%	75%					
Fixed sum for obstetric care in PSDN + referral (risk is borne by PSDN)					75% = 2250 MRO	75% = 2625 MRO	2600 MRO	2600 MRO	2600 MRO
Normal delivery in other facilities					75%	75%	75%	75%	75%
Laboratory examinations	-	50%	75%	75%	75%	75%	75%	75%	75%
Dental care	-	-	50%	50%	50%	50%	50%	50%	50%
Referral care									
Fixed reimbursement for hospitalisation	10,000 MRO	10,000 MRO	20,000 MRO	20,000 MRO	20,000 MRO	20,000 MRO	20,000 MRO	30,000 MRO	30,000 MRO
Fixed reimbursement for complicated delivery + emergency transport	5500 MRO	5500 MRO	5500 MRO	5500 MRO					
Fixed reimbursement for complicated delivery + emergency transport outside PSDN obstetric care package					5500 MRO	5500 MRO	5500 MRO	5500 MRO	5500 MRO
Caesarean section (intervention only)	-	-	100%	100%					
Caesarean section (intervention only) outside PSDN obstetric care package					100%	100%	100%	100%	100%
Ceiling for ambulatory care		5000 MRO	5000 MRO	5000 MRO	5000 MRO	5000 MRO	5000 MRO	5000 MRO	5000 MRO
Medicines in private pharmacy	-	-	50%	50%	50%	50%	50%	50%	50%
Medicines in private pharmacy for chronic disease					30%	30%	30%	30%	30%

**Table 9:** Benefits package in 2012 and amounts paid by MHO and patient

Services	Type of coverage by the MHO	% co-payment	Average amount of coverage by the MHO in MRO and (EUR)	Average amount co-payment in MRO and (EUR)
Outpatient curative care	100% above deductible	Deductible	295 (0.79)	200 (0.54)
Antenatal care	75%	25%	900 (2.4)	300 (0.8)
Flat fee for delivery in PSDN including referral	75%	25%	2600 (6.97)	900 (2.4)
Deliveries in non PSDN facilities				
Normal delivery in other health centres	75%	25%	2607 (6.98)	869 (2.33)
Referral for complicated delivery + transport	Fixed reimbursement	Above ceiling	5500 (14.74)	Unknown
C-section in Cheikh Zayed (technical act only)	Fixed reimbursement	Above ceiling	30,000 (80.4)	Unknown
C-section in CHN (technical act only)	Fixed reimbursement	Above ceiling	20,000 (53.6)	Unknown
Laboratory in first line	75%	25%	1136 (3.04)	379 (1.02)
Dental care	50%	50%	1326 (3.55)	1326 (3.55)
Hospitalisation	Fixed reimbursement	Above ceiling	30,000 (80.4)	Unknown
Ambulatory hospital services	100% up to ceiling of 5000 MRO	Above ceiling	2776 (7.44)	0
Medicines in private pharmacy	50% reimbursed	50%		
Medicines in private pharmacy for chronic care	30% reimbursed	70%		
Average amount medicines in pharmacy			821 (2.2)	Unknown

### Reluctance to change

When participative evaluation, identification of primary causes of stagnation and additional training on their management had not been sufficient to trigger change, training and support concentrated on the management cycle, specifically on evidence-based decision-making for change. Consequently Board members made good action plans that were still not put into practice. There were still other motives that hampered implementation.

At first, both MHO leaders and PSDN support team were reluctant to deviate from the original design inspired by the ILO model. The PSDN team soon understood the need to make adaptations, but the MHO leaders remained committed to the initial design. When results did not reflect expectations, tense relations between MHO leaders and PSDN also obstructed rational decision-making. The MHO leaders did not master all aspect of MHO management but did not easily take advice. Opposition sometimes seemed a way to manifest authority. Board members referred to their statutory authority when opposing change. Gradually, their conservative decisions undermined long-term viability of the MHO.

### Governance

According to the Statutes, elections should take place every 2 years to ensure rotation of members of management bodies. Such elections took place in 2005 and 2007, but the way they were organised did not allow new candidates to stand for office. The General Assembly could accept or reject a list of candidates proposed by the outgoing Board of Administrators. With minor changes, the same people were re-elected for positions in the Board of Administrators, Executive Committee, Control Committee and *bureaux de zone*. Since most of these structures were inactive – only a handful of Board members held occasional meetings – being elected for a position seemed an issue of importance.

In 2011, the supporting organisations thought that new, dynamic leaders needed to be found amongst the remaining active delegates and competent new recruits. Teachers contacted in schools and women of the centres for women’s promotion did not react positively. Many women of the promotion centres had been members in the past. Several had been discouraged when they tried to participate in leadership and none were motivated to join again. Moreover, since the network of delegates was no longer operational, communication channels with members were broken, which made it a challenge to organise elections.

Democratic members’ participation, evident during early meetings of the General Assembly, was reversed in subsequent years. Voting practice was regular and the majority vote respected, but the information given on which to base the vote was not always objective. The agenda changed from discussing and deciding priority issues to communications by predominant community leaders. The last annual meeting of the GA took place in 2009. Members could still voice their opinion during the annual sensitisation campaigns, but these events were not conducive to productive exchange. Members expressed grievances without listening to answers, while the attitude of the MHO leaders was defensive rather than objectively considering the problem advanced.

In the end, a small group of leaders monopolised decision-making. Also the manager lost her spirit of innovation and executed routine tasks she would no longer question. The MHO design and procedures meant to promote greater participation, did not introduce new interactions between members. In fact, the opposite happened: the power structures and decision-making processes existing in the community were replicated in the MHO management.

### Social and ethnic tensions

Many of the attitudes that underlie the actions of the MHO leaders, and of all stakeholders, have their roots in traditional social order. Leadership is usually the domain of people of Maure origin, in society as in government institutions. For the members of the Board of Administrators of the MHO, predominantly of Pular origin, the MHO is an independent organisation that presents a rare opportunity to exert leadership. Defending their leadership and independence may explain why they stayed away from cooperation with other organisations. For example, “the imperative to safeguard the autonomy of the MHO” justified their refusal to engage with local authorities for greater integration of the MHO into the socio-political structures of Dar Naïm. Hierarchical structures in society also underlie their reluctance to approach hospital physicians and discuss rational prescription.

Socio-economic belonging also influenced recruitment strategies. Most members of the MHO belonged to the poorer strata of society. Why recruitment among the wealthier people was rarely attempted was never clearly explained. Formal sector workers, estimated at 30% of the population of Dar Naïm in 2009, were covered by the National Health Insurance Scheme (CNAM) for 90% of hospitalisation fees, but they might have found coverage of the remaining sum and of first line care useful. Other higher income groups such as shopkeepers and taxi drivers had also not been approached.

A remark made in 2009 brought into attention the tensions between inhabitants of Dar Naïm, and their impact on the expansion of the MHO. When discussing low membership in the zones *Eolienne* and *Tensouïlim* (Table 6), the MHO leaders explained that spreading information had been insufficient because they did not know anyone there. After 9 years of MHO promotion, this argument did not seem credible. In fact, campaigning in these zones had been avoided because of the unease to approach people from different ethnic and socioeconomic background who live there. In addition, when in 2011 the PSDN recommended renewed efforts to attract the merchants, civil servants, health personnel, teachers and other professionals, members of the Board were aware that those new recruits could threaten their authority in the MHO and their position in the Board of Administrators. Instead, the Board of Administrators focused their recruitment efforts on those zones where they felt in-charge.

### Layers of primary causes and underlying drivers

Reluctance to implement needed changes was the main obstacle towards expansion of the MHO of Dar Naïm. This manifested itself in unwillingness to deviate from the initial strategies even when not well adapted to local circumstances, and in not engaging in planned activities in the fields of resource mobilisation, cost containment, cooperation and negotiation with partners, financial management, communication with members, members’ retention and ultimately sustainability of the MHO.

Why the planned changes were not implemented can be explained by a combination of underlying grounds. Their insufficient preparedness for the task made it difficult for the members of the Board of Administrators to take adequate decisions. This should not have been an obstacle if they had been more open to training or advice on technical issues. Why they overruled advice had much to do with power relations, social status and personal fulfilment. Tensions about who was in charge sometimes overshadowed rational decision-making. The suggestions of the knowledgeable but female manager were usually not taken on board. Discussing cost containment with hospital physicians who had higher social status was avoided. Implementing change was perceived as proving original decisions wrong and therefore losing face. The MHO decision-makers withdrew into routine activities without recognising the long-term consequences of their decisions for the viability of the MHO. Their desire to remain in charge prevented other-minded delegates to join the Board of administrators. Ethnic and socio-economic tensions, finally, determined relations between actors and prevented inclusive recruitment.

## Discussion

The objective of this study was to understand why the MHO of Dar Naïm, set up in apparently favourable circumstances, did not achieve its potential. In this article, findings were organised around the outcome of membership. Like in most studies focusing on reasons for low membership, factors contributing to drop-out and low subscription were easily identified. Unlike most studies, our long-term involvement in the project gave us the opportunity to look into ensuing implementation. The focus shifted from, first, the design and implementation of technical aspects to, second, overall management practices and, third, underlying factors that hampered action. The next sections briefly set our findings against those of other studies past and present.

### Looking into strategies and their implementation

Looking into the details of design and implementation processes was often the focus of early reports of CBHI programmes [2, 4, 8, 36, 37, 45] but seems now disregarded. There is reason to bring the nuts and bolts back into focus to question the effectiveness of certain procedures that are routinely put in place. For example, early reports recognised arrears in the collection of contributions as a widespread problem affecting financial viability of CBHI in West Africa [2, 9]. Yet delays in premium payment were still observed in a recent study covering several schemes in Senegal [46]. Like in Dar Naïm they identified premium collection by volunteers who had other professional duties and who had to use their own resources to pay for transport as contributing factors for high drop-out. Nonetheless, the monthly collection by volunteers remained the routine collection method.

For the leadership of the MHO of Dar Naïm, the fact that similar procedures were unchallenged in CBHI schemes in neighbouring Senegal was a more potent argument for status quo than analysis of local results was an argument for change. Yet our findings show that there is scope for improvement when specific design and implementation issues are addressed: simplifying procedures for proving entitlement improved members’ satisfaction, shifting tasks from volunteers to the paid manager improved premium collection, regular and organised platforms for communication with members reduced drop-out, and in the case of the health equity fund, seeking opportunities for tapping existing subsidies reduced the bill for the scheme.

### Overall management practices

Also the capacity to manage CBHI schemes was already discussed in early reports [2, 8, 47–49] and has been often repeated since [5, 49, 50]. Observed deficiencies in skills and knowledge are itemised for training: determination of contributions and benefit package, containing adverse selection, collection of contributions, accounting, marketing and communication, etc. The experience of Dar Naïm highlights a further need for more general management skills, such as using data produced by routine monitoring for planning, and, most importantly, management of change. The wide range of skills and knowledge required to reach high membership points at the need for skilled professional management instead of habitual voluntary management [3, 5, 51]. Skilled professional management requires adequate remuneration, which can usually not be paid from members’ contributions. A workable solution sought by many CBHI schemes is that members’ contributions cover the health care expenses, while government or external subsidies cover management expenses. Many governments and external donors, however, still insist on financially self-sufficient CBHI schemes, a strategy that inevitably translates into unpaid management and low membership.

Yet the MHO of Dar Naïm *did* have a competent professional manager, able to execute most tasks and eager to learn new skills when needed. Her action was thwarted by the decisions of the Board of Administrators, which in turn points at the crucial role of governance and the underlying social processes that rule it.

### Governance

Governance of an MHO is characterised by decision-making by the members and their participation in management [2]. Several hypotheses underlie the endorsement of active community participation as a determinant of membership. Well informed members would understand and accept the principles of risk-sharing and the mechanisms put in place to avoid threats such as adverse selection, overconsumption and abuse [8]. Transparency, accountability to members and participation in decision-making would enhance trust in scheme management [52]. Voluntary participation in management would reduce costs and make the premium more affordable [46]. Social control would reduce misuse and hence reduce costs [42]. Scheme members joining hands would have more power to influence quality of care and prices and in this way make affiliation more attractive [53–55].

The positive influence of members’ involvement on membership was illustrated in our findings by the example of the zone of *Zaatar*. Main factors were access to information, a feeling of belonging and mutual aid to pay the premium. Other studies also show the positive influence of participation on membership [13, 52]. Enrolment in the CBHI of Kisiizi in Uganda, for example, increased when members were consulted for defining the future orientation of the scheme [4]. More recently, a study in Senegal showed that the more active the mode of participation, the lower was drop-out and, on the other hand, people who dropped out were less inclined to actively participate, to trust management or to endorse the principles of risk-sharing [19].

Overall, however, community participation did not live up to its promises in the MHO of Dar Naïm. The need to actively promote change had been overlooked in governance like it was in technical management issues. In the same way as it was assumed that adequate technical design would automatically lead to expected results in terms of membership or social protection, it was assumed that carefully prepared organisational procedures would lead to participatory decision-making, without sufficient attention to implementation itself.

### Relations with external partners

Early studies discussed the relative weakness of CBHI schemes in their relationship with providers. Either they don’t have sufficient knowledge to negotiate the most advantage payment mechanisms [2], or they lack authority to influence supply [36]. Only a minority of schemes effectively engaged in any strategic purchasing activities [37]. In West Africa especially, relations between patients and health professionals were unequal [56] and a change in the balance of power between purchaser and provider may be difficult to obtain.

Two recent studies suggest that little has changed. In CBHI schemes in Benin, negotiation with health workers of the local health centre was possible but CBHI leaders had little influence at the higher levels of the health system [57]. In Senegal, MHOs seeking to negotiate conditions with hospitals had little bargaining power because of their relative insignificance for hospital finances [46].

Similar results are reported from Indian CBHI schemes. A study investigating whether and how the common action of members as a group influenced access to quality health care identifies four mechanisms by which CBHI could in theory induce providers to improve services: (1) ‘voting with the feet’, (2) imposing rules through strategic purchasing, (3) put pressure on the authorities to regulate and enforce regulations, and (4) transforming the power imbalance at the provider-patient interface [55]. In practice, strategic purchasing mechanisms such as contracts with providers were put in place, but most schemes lacked the capacity to enforce compliance. The number of hospitalised members was too insignificant to have a voice. Influencing behaviour of hospital physicians, quality standards, prices and accountability mechanisms therefore relied on higher authority in the health system that can hold the hospital management to account. But members had no influence on regulation or its enforcement in practice.

The fourth mechanism of the theoretical framework, transforming the power imbalance at the provider-patient interface, requires a radical transformation of attitudes: from the providers a more respectful and less paternalistic approach towards the poor, and from the patients an effort to become active participants in their health.

This change in power relationships is a learning process for both patients and providers [53] that, in Dar Naïm, was not even started. The MHO did not realise its theoretical potential to lobby as a group of users or to empower members in their dealings with health professionals, local authorities and other partners. The social capital framework discussed in the next section may be helpful to further explore the issue of power relations in the context of CBHI.

### Reasons for low membership from the point of view of social capital theories

A conceptual framework developed in 2008 to analyse reasons for low CBHI membership from the point of view of social capital theories [58] looks at bonding and bridging social capital at micro and macro level. At micro-level, bonding social capital, or ties within communities, creates the indispensable trust and solidarity among scheme members, but schemes that do not go beyond such close ties may remain too small for effective risk pooling or negotiation power. To reach larger membership, inter-community bridging relations need to be built to form an inclusive scheme uniting people from various communities or form federations where members prefer to identify with their basic unit [40, 49, 59].

Social capital at macro level concerns relations with government agencies and other institutions meant to assist local initiatives, with attention for the institutional capacity to provide the technical, legal, political and cultural environments required for development of the schemes [59] and direct individual relations with representatives of these institutions that enhance cooperation.

This framework was applied to CBHI schemes in Senegal where social capital was investigated as a determinant of membership [60]. Bridging social capital at local level was measured by membership of other community associations to which people belonged, and indeed, households enrolled in CBHI were significantly more likely to be members of other associations than non-member households. Also in Dar-Naïm, MHO members were more frequently member of other associations than non-members [22], but these networks were within a restricted community, did not transcend ethnic and social boundaries, and should rather be seen as intra-community networks creating bonding social capital. Efforts to strengthen bonds within the restrained community can be recognised in the decision to maintain the ineffective *bureaux de zone* in order to give all informal leaders a position of responsibility. On the other hand, the reluctance of the MHO leadership to build bridges to other communities is seen in their unwillingness to promote membership of the wealthier and people of Maure origin. CBHI membership in Dar Naïm suffered from the negative effects of bonding social capital, where strong intra-group ties, not compensated by inter-community networks, exclude other communities and engender isolation [58, 59]. Also at macro-level, the social fragmentation of the society was an obstacle to state/society bridging relations and to ties with individual representatives of these institutions.

### Limitations

Like for any case study, we cannot readily draw conclusions that universally apply. The specific underlying drivers uncovered in Dar Naïm are highly contextual. Applying the framework for analysis to other CBHI schemes and cross-case comparison may however enrich theory-building. Doing that is in turn limited by the substantial amount of data needed to explore a range of aspects over time, and by the time the analysis itself takes.

The researcher doing this analysis was the same who carried out the successive programme evaluations since 2003. Whether this is a limitation can be argued: on the one hand there may be a greater risk of bias, on the other hand, in-depth knowledge of the project may be an asset.

## Conclusions

Membership of the MHO of Dar Naïm remained far too low to consider CBHI as a promising strategy on the path towards universal health coverage in Mauritania. Taking into consideration the current knowledge on CBHI in Africa, three lessons from the analysis of the Dar Naïm MHO implementation process seem relevant for any CBHI programme that would have the ambition to extend coverage to populations earning a living in the rural and informal sector: the imperative to have a skilled professional management, the need to question indiscriminate applications of so-called ‘standard’ procedures, and the need to take context into account in all phases of the design, implementation and evaluation of CBHI.

### Competent management

Setting up and managing a CBHI scheme that reaches high membership is a complex intervention that needs trained professional management, competent in a range of skills. Training should cover all aspects of CBHI management, from technical procedures such as costing of services and membership administration, to overall management issues including problem solving and management of change, and capacity to analyse broader societal issues and interpersonal relations. Countries that otherwise promote CBHI as a strategy on the path to universal coverage do not usually avail such comprehensive training for CBHI management. Skilled management requires adequate remuneration, which will need subsidies.

### Review ‘standard’ procedures

Unsatisfactory performance of CBHI is known, low membership seems accepted as a characteristic, and still regionally prevalent models are being routinely, even rigidly, implanted without much questioning, and produce the same modest results, again unquestioned. Instead, design, procedures and management style should be revised with creativity, and sound implementation itself given more chances. If a feasibility study is done, its results should guide adequate choices. Evaluation should go beyond results but critically investigate adequacy of design and implementation to reach the expected results. Also whether some hypothetical results attributed to CBHI are at all realistic should be questioned.

### Context truly matters

Our analysis using a systems approach brought to light the overwhelming influence of social relations on decision-making, management practices and behaviour of the MHO leadership. Elsewhere, totally different underlying forces could be at play that influence results in the same way. It may be a crucial role of national or regional support centres to identify these drivers and accompany local management through the hurdles.

## Abbreviations

CBHI: Community-based health insurance
CNAM: National Health Insurance Fund (*Caisse Nationale d’Assurance Maladie*)
DAS: Department of Social Affairs (*Direction des affaires sociales*)
EUR: Euro
HEF: Health Equity Fund
ILO: International Labour Organisation
ITM: Institute of Tropical Medicine
MCSDN: Mutual Health Organisation of Dar Naïm (*Mutuelle communautaire de santé de Dar Naïm*)
MHO: Mutual health organisation
MRO: Mauritanian Ouguiya
PSDN: Projet Santé Dar Naïm
STEP: Strategies and Techniques against Social Exclusion and Poverty programme

## Footnotes

1 Caritas Mauretanie (www.caritas.org/where-we-are/middle-east-north-africa/mauritania)

2 Now APSDN: Association pour la Promotion de la Santé à Dar Naïm (http://apsdn.org/)

3 Memisa (http://memisa.be) provided financial support for the set-up and management of the PSDN, and subsequently the Mutual Health Organisation and the Health Equity Fund.

4 Institute of Tropical Medicine, Antwerp, Belgium (http://www.itg.be)
